# Assessment of Pharmacovigilance Across University Hospitals in Morocco

**DOI:** 10.1007/s40264-025-01517-w

**Published:** 2025-02-12

**Authors:** Hind Hamzaoui, Anna Shaum, Imad Cherkaoui, Latifa Ait Moussa, Houda Sefiani, Ismail Talibi, Ghita Benabdallah, Omar Salman, Seth Ferrey, Rachida Soulaymani Bencheikh

**Affiliations:** 1 Centre Anti Poison et Pharmacovigilance du Maroc, Ministry of Health, Rabat, Morocco; 2 US Centers for Disease Control and Prevention, Atlanta, USA; 3 Private Consultant, Rabat, Morocco

## Abstract

**Introduction:**

Despite the increased scrutiny on vaccine safety following the coronavirus disease 2019 (COVID-19) pandemic, Morocco’s Centre of Antipoison and Pharmacovigilance (CAPM) remained concerned that the pharmacovigilance system in Morocco was insufficiently implemented, including limited adverse event (AE) reporting, poor data use, and inconsistent training nationwide.

**Objectives:**

We sought to assess the status of pharmacovigilance activities (PAs) prior to formally institutionalizing them across university hospital centers (UHCs), given their position as the main providers of healthcare in Morocco and key sources for reporting serious AEs.

**Methods:**

We assessed seven UHCs (housing 30 hospitals) in 2023 using a structured questionnaire with pharmacovigilance focal points developed from the World Health Organization’s indicators of pharmacovigilance and the Global Benchmarking Tool. Data were grouped into 28 PAs and scored from 0 (not implemented) to 3 (fully implemented). We then calculated an implementation rate for each site on the basis of percent of PAs fully implemented (≥ 70%, well established; > 40% to < 70%, partially implemented; and ≤ 40%, not implemented). A desk review was also performed at the sites. Using the results of the assessment, three working groups of pharmacovigilance stakeholders developed recommendations to be formally adopted by UHCs.

**Results:**

Basic elements of pharmacovigilance (notification forms and VigiFlow^®^ or Excel databases) were present at all the UHCs assessed. In total, 14 hospitals (47%) had well-established PAs, including advanced activities such as signal detection of adverse events following the use of medicines and vaccines, as well as causality assessment; 9 hospitals (30%) were partially implementing pharmacovigilance, and 7 hospitals (23%) had no established activities or very basic activities. Within four UHCs, activities had not been implemented at the same level from one hospital to another and vaccine vigilance was largely deprioritized. The working groups made recommendations for improving collaboration, standardizing procedures, and outlining a new organizational structure for pharmacovigilance, which was institutionalized by a formal agreement among UHCs in July 2023.

**Conclusions:**

The assessment revealed a subgroup of centers with well-established AE signal detection and causality assessment abilities, which could play a leading role in the country. After the site assessment, our collaborative approach of bringing together focal points to identify next steps and generate buy-in helped to formalize pharmacovigilance across centers.

## 1 Introduction

The World Health Organization (WHO) defines pharmacovigilance as “the science and activities relating to the detection, assessment, understanding and prevention of adverse effects or any other possible drug-related problems” [1]. Pharmacovigilance has since widened to include other healthcare products, including vaccines, contraceptives, medical devices, blood products, and herbal products [2]. Adverse events (AEs) are composed of both drugs [adverse drug reactions (ADR)] and vaccines [adverse events following immunization (AEFI)], and reports are sent to appropriate regional centers and to the national pharmacovigilance center where this information is processed and forwarded to the WHO’s individual case safety report database—VigiBase^®^—for collation, analysis, and evaluation.

The thalidomide tragedy in the early 1960s was a wakeup call for most countries to start establishing national pharmacovigilance systems. This disaster also led to the formation of the WHO Programme for International Drug Monitoring (PIDM) in 1968 by ten pioneering members from high-income countries. Currently all high-income countries are members of the WHO PIDM [3]. The sophistication of the pharmacovigilance reporting systems varies from basic capacities in low- and middle-income countries (LMICs) to more advanced technology in high-income countries [2].

Countries are encouraged by the WHO to regularly assess and strengthen their pharmacovigilance systems for medicines and vaccines. Following the development and the adoption in 2010 of the first global indicators for assessing pharmacovigilance activities, which were classified in three groups for this purpose (structure, process, and outcome indicators) [2, 4], the 2014 World Health Assembly resolution WHA67.20 established the strengthening of regulatory systems as a global public health priority [5]. The WHO Global Benchmarking Tool (GBT) for assessing all regulatory functions, including vigilance, was thereafter implemented [6]. It was complemented by a series of performance evaluation processes (PEPs) to give a more detailed picture of how the regulatory system delivers the desired regulatory outcomes [7].

In Morocco, the first pharmacovigilance activities began in the 1980s, led by the faculty of medicine at Casablanca University [8]. In 1989, the Ministry of Health set up the central pharmacovigilance organization known today as the “Centre Anti Poison et de Pharmacovigilance du Maroc” (CAPM) [9, 10]. Among African countries, Morocco is a pioneer in international collaboration and training in the field of pharmacovigilance. It was the first African country, with South Africa, to join the WHO PIDM in 1992 [3], and the second African country after Ghana to be appointed as a WHO collaborating center for pharmacovigilance in 2011 [11]. Despite these accomplishments, CAPM remained concerned that the pharmacovigilance system in Morocco was insufficiently implemented, including limited AE reporting, poor data use, and inconsistent training nationwide.

Following the increased scrutiny on vaccine safety following the COVID-19 pandemic [12], CAPM sought, in 2022, to strengthen and institutionalize pharmacovigilance activities across the university hospital centers (UHCs), given their position as the main providers of healthcare in Morocco and key sources for reporting serious AEs, along with the pharmaceutical industry. UHCs are large public institutions in charge of care, teaching and research, and oversight of publicly provided healthcare in the 12 administrative regions of the country. UHCs in Morocco represent about 30% of the public functional bed capacity and 26% of hospital admissions in the public sector [13]. They are reference hospital structures for the public sector, and to a lesser degree, for the private sector. Among the 1729 ADR cases notified to CAPM in 2022, with an identified notifier, 66% were from the pharmaceutical industry and 19% from the UHCs. The remaining 15% of overall notifications came from other public hospitals, primary health centers, clinics, and private medical practices, as well as pharmacies or health institutes [14]. CAPM’s strategy of focusing primarily on the university hospitals to strengthen the national pharmacovigilance system dates back to 2018 with the signing of the first partnership agreement aimed at identifying pharmacovigilance structures specific to the university hospitals [15].

The objectives of this study were to assess the status of UHC pharmacovigilance activities to formally institutionalize them and to share lessons learned from the assessment with other LMICs looking to strengthen pharmacovigilance.

## 2 Methods

### 2.1 Hospital and Respondent Selection

In January 2023, we conducted a national assessment of all seven UHCs in the country, each of which comprises a group of 1–10 university hospitals in the same city, coordinated by an administrative direction (Table 1). At the time of the assessment, there were five functional UHCs and two UHCs under construction. All UHCs, even those partially functional or nonfunctional, have pharmacovigilance focal points among pharmacists and professors in pharmacology and were included in the study. The focal points are the coordinators of pharmacovigilance activities, including reporting AEs within the UHC hospitals (Table 1).

### 2.2 Data Collection

We developed an assessment questionnaire to evaluate the pharmacovigilance system within the UHCs (Online Supplementary Material 1). The questionnaire was prepared using the indicators from the WHO Pharmacovigilance Indicators Manual [2] and the WHO Global Benchmarking Tool (GBT) [6], as well as by adapting the Field Visit Manual for Assessing the Performance of Vigilance Function to the local context for health-facility-level assessment [6]. It was designated to collect information about pharmacovigilance activities related to medicines and vaccines supported by the UHCs. The questions focused on six areas of pharmacovigilance recommended by the WHO in the vigilance field visit assessment questionnaire [7]: (1) systems, structure, resources, and stakeholders; (2) case detection, notification, and management; (3) analysis of ADR cases; (4) analysis of AEFI cases; (5) adverse events signal detection and evaluation; and (6) training, information, education, and communication. A total of 43 questions were established, including both closed- and open-ended questions (Table 2).

The questionnaire was piloted during an informational session with the UHCs in Rabat in December 2022 to prepare the participants for the field assessment. The assessment visits were conducted between 5 and 24 January 2023 by a team of CAPM staff and 2 CDC experts. For each UHC, the assessment team visited the UHC direction at the assessment sites and conducted in-person interviews with the pharmacovigilance focal points to complete the questionnaire. At the UHC Direction, a desk review of pharmacovigilance-related documents was conducted where available, including AE data from each UHC (which includes the hospitals within that UHC) from 2020, 2021, and 2022. The results were reviewed and subsequently entered in an Excel database.

### 2.3 Data Analysis

We used the recommended methodology of the WHO national vigilance systems assessment as the basis of the analysis [2, 6, 7]. The data from the questionnaire were grouped into 28 pharmacovigilance activities representing the 6 abovementioned areas of pharmacovigilance, which were then summarized descriptively.

The 28 pharmacovigilance activities were categorized into four scores according to the WHO recommendations [6]:

- 3 points: activity or group of activities FULLY implemented;
- 2 points: activity or group of activities PARTIALLY implemented;
- 1 point: activity or group of activities MINIMALLY implemented; and
- 0 points: activity or group of activities NOT implemented.

We then considered an implementation rate (IR) for each site assessed as the proportion of the 28 assessed activities that were fully implemented. It was calculated as the sum of the observed scores of the 28 pharmacovigilance activities divided by the theoretical maximum of 84, if all 28 activities were fully implemented (84 = 28 × 3).


$$\text{IR}=\frac{\text{Observed}\;\text{score}\;\left(\text{activity}\;1\right)+\text{Observed}\;\text{score}\;\left(\text{activity}\;2\right)+\cdots +\text{Observed}\;\text{score}\;\left(\text{activity}\;28\right)}{84}\times 100$$

Following the IR, we then further classified hospitals and UHCs into three final levels:

- Level 1: well-established pharmacovigilance (IR met or exceeded 70%);
- Level 2: partially implemented pharmacovigilance (IR > 40% but < 70%); and
- Level 3: basic or no pharmacovigilance (IR ≤ 40%).

### 2.4 Data Review and Working Groups

Following the assessment, we sought to consensus-build a new structure of pharmacovigilance across Morocco’s UHCs, in hopes of then formally institutionalizing it through UHC internal regulation. For this purpose, we set up three thematic working groups with representatives of pharmacovigilance from both the CAPM and all the UHCs, including the pharmacovigilance focal points and other local stakeholders. Each working group was organized and tasked with reviewing the materials from the assessment related to one of the following topics: (1) implementing a new pharmacovigilance structure within UHCs, (2) updating the procedures, and (3) establishing a means of collaboration within and between UHCs. From March to April 2023, each working group developed recommendations in their specific activity area through virtual meetings, and the three working groups held a face-to-face restitution meeting, at a central level, in May 2023.

## 3 Results

At the time of the assessment, six of the seven UHCs were functioning fully or partially (Agadir UHC was functioning partially and the UHC in Tangier was set to open later in 2023). At the UHC level, 18 pharmacovigilance focal points or their representatives were interviewed, including 17 (94%) belonging to the 6 functional UHCs (Table 1). In total, 12 (67%) of the focal points were pharmacists working in a hospital or central pharmacies, of whom 9 (50%) were head pharmacists, and 2 focal points (11%) were professors of pharmacology in charge of pharmacovigilance and toxicology laboratories that were located outside university hospitals, either in the Faculty of Medicine and Pharmacy or in a clinical research center of the UHC. Finally, one (6%) focal point was both a head pharmacist and professor of pharmacology, and three (17%) were professors of pharmacology. At the hospital level, the focal point from only one UHC reported and documented having officially designated pharmacovigilance contact persons in all clinical wards. In the other UHCs, the deployment of pharmacovigilance contact persons in clinical wards was nonexistent or exceptionally limited to a few university hospitals, in an informal way. Desk reviews were carried out at the 6 functioning UHCs and at 19 of the 30 hospitals. Our assessment showed different levels of implementation of pharmacovigilance activities and collaboration between the CAPM and UHCs from one UHC to another, as well as from one hospital to another within the same UHC (Tables 3 and 4).

### 3.1 Description of Pharmacovigilance Activities Across UHCs (*n* = 7)

At the UHC level, focal points reported that for most UHCs (*n* = 6; 86%), although pharmacovigilance activities did not have an annual budget in the hospitals, they were included within the hospital’s strategic operating plan (Table 3). Focal points from four UHCs (57%) reported that they have their own procedure for implementing pharmacovigilance in the hospitals, and the AE/AEFI notification form was used across all UHCs (100%). The presence of a database (either VigiFlow^®^ or Excel) or an archive of pharmacovigilance cases was found within all the UHCs evaluated (100%). Focal persons reported that investigated cases had undergone causality assessment in only two out of the seven (29%) UHCs assessed. However, even if pharmacovigilance activities were reported to be present at the UHC level, they may not have been deployed in all the hospitals in the UHC, particularly within the largest ones, which have several officially designated focal points (Table3). Focal points reported that four out of seven (57%) UHCs had a regular collaboration with the CAPM to review safety issues for medicines and other health products and that there was an established notification route for reporting suspected AE cases to the CAPM in most UHCs (*n* = 6; 86%) (Table 4). Focal points reported that five out of the seven UHCs (71%) participated in training sessions and pharmacovigilance scientific days^1^ organized by the CAPM during the previous 2 years (2021–2022) (Table 4). The collection of ADR data was consistent and continuous during the period investigated from 2020 to 2022 for only three (43%) of the seven assessed UHCs. However, the comparison of ADR data available locally and those received at the CAPM showed that ADR reporting to the central level (CAPM) was only significant for two of these three UHCs. Notification of AEFIs not associated with COVID-19 vaccines was nonexistent at the local level during this period. The transmission of AEFIs associated with COVID-19 data to the central level (CAPM) was done through a specific circuit set up for all hospitals, including UHCs, during the pandemic (Table 5).

### 3.2 Description of Pharmacovigilance Activities Across Hospitals in the Seven Assessed UHCs (*n* = 30)

At the hospital level, most pharmacovigilance activities were widely implemented (more than 70% of the UHC hospitals), such as the inclusion of pharmacovigilance within the annual strategic operating plan of hospitals or the availability of the standard national AE reporting form (Table 3). However, pharmacovigilance activities such as e-reporting, AEFI notification, causality assessment of investigated cases, or knowledge of notification deadlines (particularly for AEFI) were poorly implemented (fewer than 30% of the UHC hospitals were implementing the activity) (Table 4).

### 3.3 Final Categorization of Hospitals and UHCs by Level of Implementation of Pharmacovigilance Activities

Following analysis, we were able to categorize the UHCs and hospitals into three groups according to their level of implementation of pharmacovigilance activities (Online Supplementary Material 2):

Level 1: In total, 14 hospitals (47%) belonging to 3 UHCs had well-established pharmacovigilance activities; their implementation rates met or exceeded 70%. Almost all of the strategic pharmacovigilance activities were present in this group of university hospitals. This group had all the key pharmacovigilance activities such as dissemination of national guidelines to hospital staff, establishment of procedures, inclusion of pharmacovigilance in hospital strategic plans, notification of all types of pharmacovigilance problems, feedback to internal notifiers using several methods, multiplicity of mechanisms for disseminating pharmacovigilance information to hospital staff, and intra-hospital continuous training in pharmacovigilance. They also had advanced levels of pharmacovigilance activities such as signal detection and validation, as well as decision-making following adverse event concerns. This group included the only UHCs that reported investigations of serious AEFI cases.

Level 2: In total, nine hospitals (30%) belonging to three UHCs were partially implementing pharmacovigilance activities and were working to better establish the activities in their institutions; their implementation rates were less than 70% but not below 40% and they had the basic operating elements of pharmacovigilance (reporting forms and access to VigiFlow^®^). Reporting was restricted to only certain types of pharmacovigilance, and the mechanism for disseminating pharmacovigilance information consisted almost exclusively of information letters issued by the hospital leadership.

Level 3: In total, seven hospitals (23%) belonging to three UHCs had no established or very basic pharmacovigilance activities occurring. This group included UHC hospitals that were not very collaborative or still nonoperational. The implementation rates of strategic pharmacovigilance activities in these structures remained below 40%.

For the UHCs and hospitals assessed as “level 1: well-established pharmacovigilance,” many AEs were reported by the sites during recent years (2020–2022). Underreporting to the central level, as identified by a comparison between individual UHC data and data from the national database at the CAPM, was observed in two of the four UHCs from level 1. Furthermore, the collection of pharmacovigilance data was only correctly reported in two hospitals out of ten within the largest UHC in Morocco, which is located near the CAPM and benefits from close collaboration. For the UHCs and hospitals that met level 2, AE data were only limited to a few dozen cases reported annually. For the group of hospitals and UHCs that met level 3, reporting was nonexistent. Almost all cases were reported following COVID-19 vaccination, highlighting a substantial gap in AEFI reporting for routine immunization (Table 5).

### 3.4 Postassessment Working Groups

Following the assessment, the three thematic working groups developed recommendations to address the gaps identified in the results. Working group members included both central and local pharmacovigilance stakeholders. Members of each working group included a CAPM coordinator as the group leader, with two key members of the project steering committee and seven pharmacovigilance representatives from the UHCs. Additional resource persons from the CAPM officially attended the working group meetings and the UHC representatives invited the assistants they deemed necessary.

**Fig. 1:**
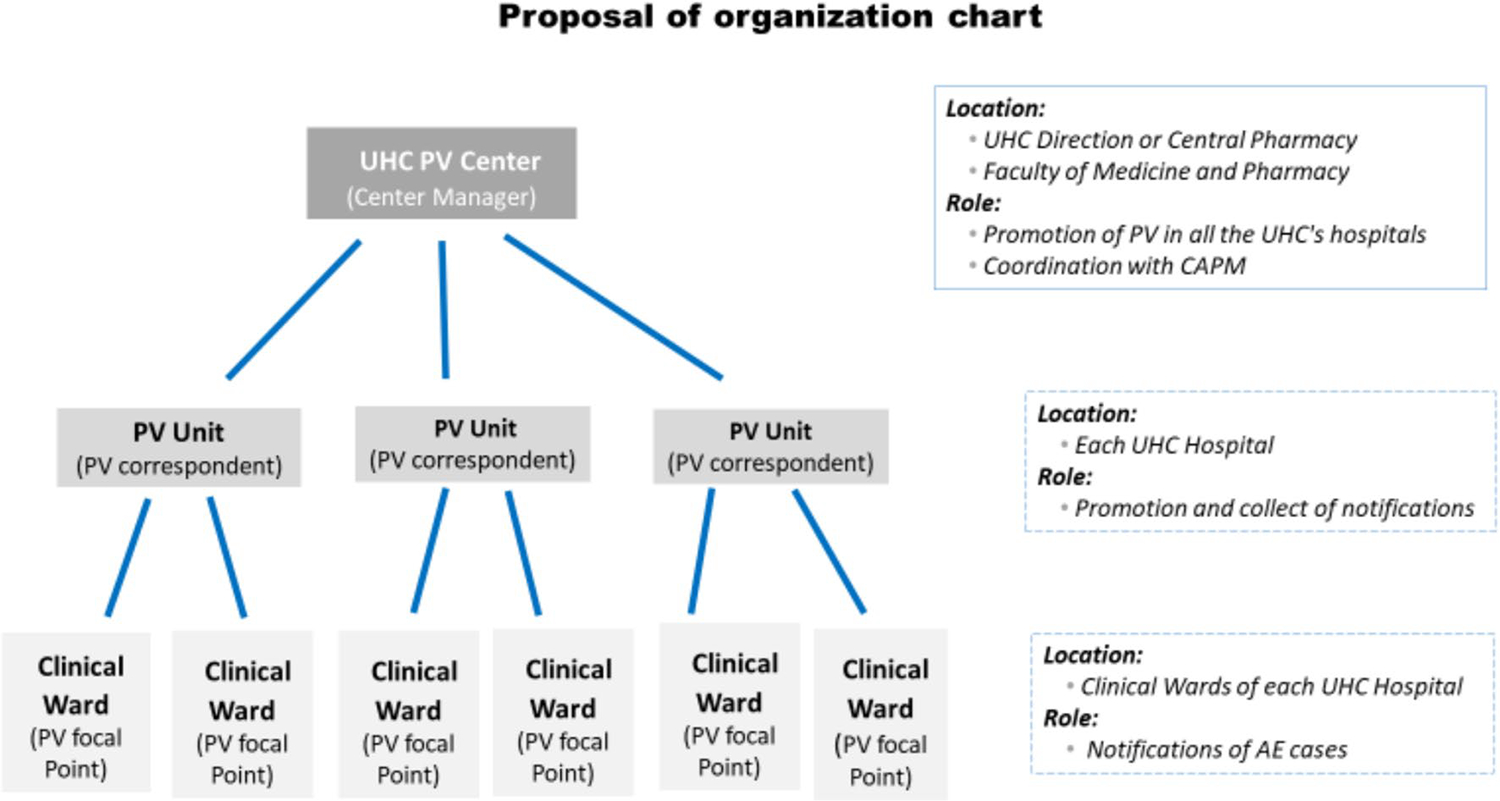
Organization of pharmacovigilance (PV) at the university hospital center (UHC)

The first working group developed an organizational and structural chart (Fig. 1) for pharmacovigilance and highlighted the following elements:

- a pharmacovigilance center in every UHC with a center manager responsible for the promotion and functionality of pharmacovigilance and coordination with the central level (CAPM);
- a pharmacovigilance unit in each university hospital under the authority of the hospital direction, with a pharmacovigilance correspondent responsible for promoting and collecting adverse event (AE) reports, as well as validating, analyzing, and forwarding cases to the center; and
- a pharmacovigilance focal point in charge of AE notification in each clinical ward of the university hospitals depending on the UHC.

**Fig. 2:**
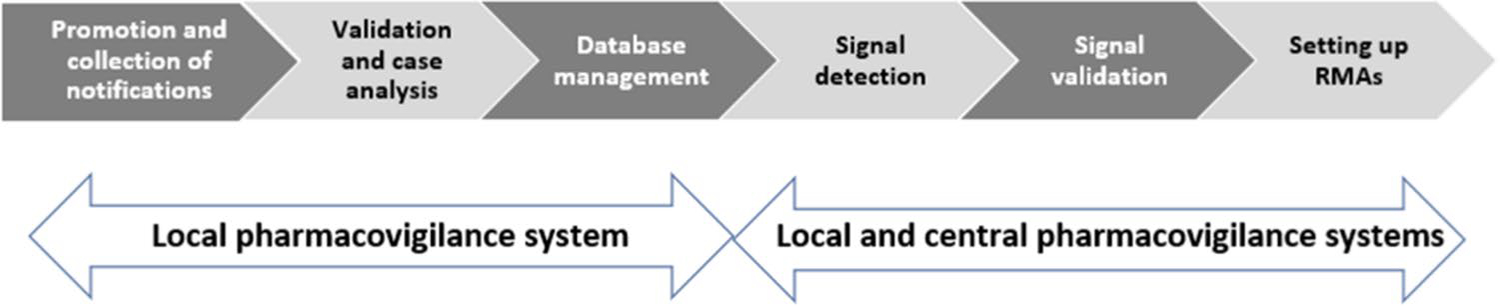
Overall pharmacovigilance process with six steps

The second working group developed guiding principles for pharmacovigilance procedures that could be standardized across all UHCs. The group first worked to reach consensus on defining pharmacovigilance in Morocco, which includes surveillance for any type of health product and any type of adverse event (expected or new, serious or nonserious). Thereafter, six steps of pharmacovigilance procedures (Fig. 2) were considered:

Step 1: promotion and collection of notifications, raising awareness and considering all categories of staff and patients as notifiers, as well as standardizing the means of notification (standard form) with a preference for “e-Reporting”;

Step 2: validation and case analysis, promoting the completion of cases and the causality assessment with feedback to the notifier;

Step 3: database management, expanding access to Vigiflow^®^ to all focal points in the UHCs;

Step 4: promotion of signal detection;

Step 5: using signal validation, setting up pharmacovigilance committees and promoting communication plans (pharmacovigilance bulletin, scientific publications, etc.);

Step 6: setting up risk minimization actions (RMA).

The third working group focused on improving collaboration in pharmacovigilance. They developed four axes of collaboration:

1. All UHCs shall enter all AEs into a VigiFlow^®^ terminal to ensure that all cases will be channeled to the CAPM VigiFlow^®^ container.
2. Since signal detection should be performed on the largest possible data pool, the UHCs should assign their signal detection resources to a collaborative activity involving all of them and the CAPM performing detection on a nationwide AE data pool.
3. Expertise sharing through regular meetings (causality assessment staff) and online sharing platforms, as well as collaboration on research projects and staff exchanges.
4. National training of the pharmacovigilance team and basic and continuing training for health professionals.
5. Communication with partners, health professionals, and patients through newsletters, pharmacovigilance review, social networks, national and international conferences, and scientific publications.

Our assessment and working group process ended in July 2023 and was concluded by the development of a new partnership agreement signed by the director of the CAPM and all UHCs. The agreement institutionalized pharmacovigilance within the UHCs of Morocco and codified the recommendations raised from the abovementioned working groups [16]. Since 2023, the UHCs have integrated the pharmacovigilance structures recommended in this agreement into their organization charts, both at the UHC administrative direction level and at each university hospital dependent on the UHC. As a result, the staff needed to run these structures were officially appointed, with ongoing training provided by the CAPM. With the Uppsala Monitoring Center (UMC), which is the WHO Collaborating Centre for International Drug Monitoring, access to Vigiflow^®^ was made available by the CAPM for each of the Moroccan UHCs. The collaborative network formed by the CAPM and the UHCs was involved in new pharmacovigilance assessments with the support of international organizations.

## 4 Discussion

It was not until the 1990s that LMICs began to join the WHO’s PIDM, but since then, their number has increased considerably in the list of members of this WHO program [3]. Pharmacovigilance systems in low-income countries differ from those with higher incomes owing to local contextual factors such as lack of healthcare infrastructure, low health expenditure, shortage of health personal, predominance of infectious diseases, and political instability [17]. Conversely, most high-income countries have robust pharmacovigilance systems with allocated budgets for pharmacovigilance activities [18].

Given this context, lessons from high-performing LMICs that are succeeding in building pharmacovigilance systems—with fewer resources than high-income countries—are greatly needed. To further strengthen Morocco’s regulatory processes, we sought to assess the growth and performance of Morocco’s pharmacovigilance systems in light of the new role of UHCs in overseeing all publicly provided healthcare in the country. The WHO pharmacovigilance indicators, with the GBT tool [6], provided a systematic approach for assessing the capacity of a country’s pharmacovigilance system.

Following our assessment, we categorized Morocco’s UHCs into three groups, according to their level of implementation of pharmacovigilance activities. The highest-performing group included almost half (47%) of the assessed hospitals with well-established pharmacovigilance activities, including AE signal detection, validation, and decision-making. This group included the two oldest UHCs, dating back more than 40 years with longstanding experience in pharmacovigilance. Historically, they played a pioneering role in the introduction of pharmacovigilance in Morocco during the 1980s. The second group was composed of UHCs and almost a third of the assessed hospitals (30%) with basic operating elements of pharmacovigilance (notification forms and access to VigiFlow^®^). The third and final group was mainly composed of the nonfunctional UHCs that opened in 2023. Within some UHCs, pharmacovigilance activities have not been implemented at the same level from one hospital to another. Our assessment also identified a few hospitals without pharmacovigilance activities or only very rudimentary ones.

Across UHCs, we identified a substantial gap in vaccine safety when compared with drug safety, as most focal points were not aware of AEFI guidelines, reporting, investigation, or causality assessment processes. Our assessment revealed that the considerable development of vaccine vigilance (e.g., increase in AE reporting) during the COVID-19 pandemic remained a temporary achievement linked to the mass vaccination during the pandemic. This is likely because the extra staff who did vigilance monitoring during mass COVID-19 vaccination efforts returned thereafter to other roles, which were unrelated to pharmacovigilance.

Over the last few decades, patient safety issues, such as underreporting, have been featured prominently on the healthcare policy agenda in developed countries. Underreporting of incidents was well described in literature, as it was considered a major handicap to a functional pharmacovigilance system, including in less developed countries such as those in Sub-Saharan Africa [19–23]. Literature has documented barriers to reporting by health professionals including the time required to report, fear of the reporting consequences, lack of feedback, and uncertainty about what to report [24]. If we exclude pandemic AEFI cases of 2021, our assessment also showed a high level of underreporting of AEs between 2020 and 2022. Underreporting of ADRs from several UHCs to the central level (CAPM), where the national database is managed, was also high despite UHC participation in the training sessions and pharmacovigilance scientific days organized by the CAPM over the previous 2 years (2021–2022). Reporting of AEFIs not associated with COVID-19 vaccines was nonexistent before and after the pandemic owing to a lack of awareness among pediatricians. In addition, the development of vaccine vigilance during the COVID-19 pandemic remained a temporary achievement linked to the institutional support accompanying the mass vaccination during the pandemic.

The first systematic review focusing on pharmacovigilance system performance in LMICs included 21 peer-reviewed studies published between 2012 and 2021, covering 51 countries from Africa, Asia, and South America [25]. The review revealed a lack of standardization regarding the methods of evaluating pharmacovigilance systems, including the WHO tools used in our assessment. However, when investigating the number of all 63 WHO pharmacovigilance indicators, the studies achieved an average use of 17.2 indicators. Our study is in line with this review, as 17 WHO pharmacovigilance indicators were used for the assessment (Table 2). This review observed that a facility for carrying out pharmacovigilance activities, a standardized ADR reporting form, and existing pharmacovigilance regulations were reported as present by most of the studied countries (92%, 86%, and 80%, respectively). However, only 63% of the countries had a pharmacovigilance advisory committee and only 35% had regular financial resources for conducting pharmacovigilance activities. A documentation of causality assessment was sparse, with only Ethiopia, Kenya, Tanzania, and Zimbabwe reportedly performing assessments.

High-performing pharmacovigilance systems in LMICs were distinguished by the presence of a specifically dedicated budget, communication tools to stakeholders such as a newsletter or website, technical assistance by an advisory committee, and the incorporation of pharmacovigilance into the national curriculum of healthcare professionals [25]. Many of these elements were identified across the UHCs in Morocco in our assessment, with the exception of special budgets.

One notable success of this project was our ability to leverage the results by setting up thematic working groups to strategize how to better implement pharmacovigilance more widely across the country. Our collaborative approach brought together multiple stakeholders, including pharmacovigilance representatives from the UHCs to share ideas and generate buy-in across hospitals. The working groups proposed a new structure of pharmacovigilance that was successfully adopted by the government and UHCs. The activities of these working groups raised awareness that the effectiveness of pharmacovigilance systems depends on their understanding of the reporting processes. For this purpose, AE data should be gathered, analyzed, and shared with UHCs. Our experience has demonstrated that good collaboration between system partners at the national level acts as a catalyst for the whole system. The development of effective networking between partners at all levels will substantially enhance scientific collaboration both at the national and international levels, in particular with other LMICs from Africa. Thus, a readiness assessment to conduct active surveillance of AEFI with the UHCs was developed by the CAPM on the basis of these project results and recommendations.

### 4.1 Limitations

We conducted in-person interviews with 18 pharmacovigilance focal points usually positioned at the administrative direction of the UHC. Most of them oversaw both the hospital pharmacy and the pharmacovigilance system within several hospitals of the UHC. Thus, responses from the hospitals at which the UHC focal points were positioned may have been more thorough than those that they oversaw in theory. Focal points may have also overestimated the amount of pharmacovigilance activities within their university hospitals owing to response bias and not revealing the work constraints linked to their double workload and staff shortages.

## 5 Conclusions

The assessment revealed that the basic operational elements of pharmacovigilance are present at almost all the sites assessed, with a subgroup of UHCs with well-established signal detection and causality assessment, which could play a leading role in strengthening the other UHCs. However, pharmacovigilance activities at UHCs in Morocco, especially on-site AEFI detection and AE transmission to the CAPM, remain suboptimal and need improvement. After the site assessment, our collaborative approach of bringing together focal points and other stakeholders across the country to identify the next steps and generate buy-in also helped to formally institutionalize pharmacovigilance at UHCs.

## Supplementary Material

supp2

supp1

## Figures and Tables

**Table 1:** Characteristics of the university hospital centers (UHC) of Morocco with the distribution of the 18 participating pharmacovigilance focal points, January 2023

Assessed UHC code (*N* = 7)	Date UHC was established	Total bed capacity	Number of hospitals (*n* = 30)	Number of pharmacovigilance focal points (*N* = 18)
1	1954	2347	10	10
2	1983	1685	4	2
3	2001	1598	5	1
4	2009	880	5	1
5	2014	673	4	2
6^a^	Not yet	867	1	1
7^b^	2023	797	1	1

a Under construction and partially functional

b Under construction and nonfunctional

**Table 2:** Types and number of indicators used in the study benchmarking questionnaire (Morocco, January 2023)

Type of indicator		Areas of pharmacovigilance					
		(1) Systems, structure, resources, and stakeholders	(2) Case detection, notification, and management	(3) Analysis of ADR cases	(4) Analysis of AEFI cases	(5) Adverse events signal detection and evaluation	(6) Training, information, education, and communication
Number of questions (*n* = 43)		12	9	4	6	5	7
WHO pharmacovigilance indicators^a^ *(n* = 17)	Core structural indicator	8					1
	Complementary structural indicator	4					
	Core process indicator		1	1			
	Core outcome or impact indicator					2	
WHO Global Benchmarking Tool (GBT)^b^ (*N* = 12)	Legal provisions, regulations, and guidelines	1					
	Arrangement for effective organization and good governance	2					
	Human resources to perform vigilance activities	1					
	Procedures established and implemented to perform vigilance activity indicators	2	1	1		2	2
Performance evaluation indicators for vigilance^c^ *(n* = 4)			2			1	1

a WHO Pharmacovigilance Indicators manual [2]

b WHO Global Benchmarking Tool (GBT) [5]

c Field Visit Manual for Assessing the Performance of Vigilance Function for health-facility-level assessment [6]

**Table 3:** Responses^a^ on key programmatic and organizational aspects of pharmacovigilance within university hospital centers (UHCs) and their respective hospitals in Morocco, January 2023

Assessment topic	Activity implemented			
	Within UHCs^b^ (*N* = 7)		Assessed UHC hospitals (*N* = 30)	
	*n*	(%)	*n*	(%)
Pharmacovigilance (PV) included within annual strategic operating plan of the hospital	6 (86)		24 (80)	
There is an annual budget component specific to pharmacovigilance in the hospital	0 (0)		0 (0)	
There is a general procedure for operating pharmacovigilance in the hospital	5 (71)		18 (60)	
The necessary tools for the operation of pharmacovigilance are available:				
AE/AEFI reporting form	7 (100)		30 (100)	
Access to VigiFlow^®^	7 (100)		23 (77)	
WHO AEFI case investigation form	4 (57)		9 (30)	
Other^c^	3 (43)		9 (30)	
The reporting structure is clear	5 (71)		22 (73)	
If the AE notification tool is in paper form, the sheet used is:				
A standard national reporting form (yellow sheet)	5 (71)		22 (73)	
Other paper form^d^	3 (43)		9 (30)	
If the AEFI notification tool is in paper form, the sheet used is:				
A standard national reporting form (yellow sheet)	4 (57)		12 (40)	
A specific WHO AEFI sheet with the 25 basic variables	3 (43)		13 (43)	
Other paper form^e^	1 (14)		1 (3)	
There is a database or archive of PV cases at the hospital level	7 (100)		24 (80)	
The type of PV database at the hospital level (multiple responses are possible):				
Excel	5 (71)		17 (57)	
VigiFlow^®^	5 (71)		15 (50)	
Paper-based register	4 (57)		10 (33)	
Other^f^	3 (43)		6 (20)	
PV case investigation reports were transmitted to the CAPM in 2021	3 (43)		10 (33)	
Investigated cases have undergone a causality assessment	2 (29)		5 (17)	

a On the basis of interviews with pharmacovigilance focal points (*n* = 18) and desk reviews conducted at UHC and hospital levels

b An activity is implemented in at least one hospital in that UHC

c Other included: one UHC with a patient file specific to the pharmacology department, one UHC with a hospital information system, and one UHC with a database (*n* = 3)

d Other included: three UHCs with adapted hospital paper forms (*n* = 3)

e Other included: one UHC with an adapted hospital paper form (*n* = 1)

f Other included: one UHC with a hospital information system, one UHC with archiving of paper forms, and one UHC with both (*n* = 3)

**Table 4:** Responses^a^ to key questions assessing collaboration in pharmacovigilance between the CAPM and university hospital centers (UHCs) in Morocco, January 2023

Assessment topic	Activity implementation			
	Within UHCs^b^ (*N* = 7)		Assessed UHC hospitals (*N* = 30)	
	*n*	(%)	*n*	(%)
There is regular collaboration between the CAPM and the hospital to review safety issues for medicines and other health products	4 (57)		17 (57)	
There is an established reporting flow for sending AE cases to the CAPM	6 (86)		21 (70)	
The means of notification used to transmit AE cases to the central level (CAPM) are:				
Notification sheet for ADR (yellow sheet)	3 (43)		12 (40)	
CAPM phone number	4 (57)		14 (47)	
VigiFlow^®^	4 (57)		14 (47)	
AEFI reporting form	3 (43)		7 (23)	
Email	3 (43)		9 (30)	
WhatsApp	2 (29)		5 (17)	
SMS	2 (29)		4 (13)	
Pharmacovigilance staff	1 (14)		3 (10)	
Excel	1 (14)		3 (10)	
Electronic notifications (e-reporting)	1 (14)		1 (3)	
Other^c^	3 (43)		5 (17)	
The notification deadlines for AEs/AEFIs/medical devices specified in the national guidelines are known:				
ADR	5 (71)		11 (37)	
AEFI	4 (57)		7 (23)	
Medical devices	4 (57)		9 (30)	
The hospital staff has participated in the training sessions and PV scientific days organized by the CAPM during the previous 2 years (2021–2022)	5 (71)		20 67)	

a On the basis of interviews with pharmacovigilance focal points (*n* = 18) and desk reviews conducted at UHC and hospital levels

b An activity is implemented in at least one hospital in that UHC

c Other included: one paper letter from hospital management, one fax, and one patient who transported its own sample to the CAPM central laboratory (*n* = 3)

**Table 5:** Comparison of number of serious adverse event reports observed during pharmacovigilance (PV) desk reviews at university hospital centers (UHCs) compared with the number of reports received in the CAPM national database (Morocco, January 2023)

UHC code (*N* =7)	Number of hospitals (*N* = 30)	Implementation level of PV^a^	Total reported ADRs						Total reported AEFIs not associated with COVID-19 vaccines						Total reported AEFIs associated with COVID-19 vaccines						
			Data reported by UHC			CAPM national database (VigiFlow^®^)			Data reported by UHC			CAPM national database (VigiFlow^®^)			Data reported by UHC			CAPM national database (VigiFlow^®^)			CAPM specific database (COVID-19)^b^
			2020	2021	2022	2020	2021	2022	2020	2021	2022	2020	2021	2022	2020	2021	2022	2020	2021	2022	2021
1	10	1, 2, 3	139	189	218	159	206	315	0	0	0	0	0	0	0	726	0	0	697	0	10132
2	4	1,2	347	127	372	56	17	102	1	0	0	0	0	0	0	406	20	0	1	15	12120
3	5	2	120	6	21	5	31	14	0	0	0	0	0	0	0	106	0	0	0	0	3247
4	5	1	84	60	84	70	56	83	0	0	0	0	0	0	0	393	1487^c^	0	6	4	3226
5	4	1	29	24	39	0	0	0	0	0	0	0	0	0	0	32	0	0	0	0	2032
6	1	2	5	6	15	2	0	1	0	0	0	0	0	0	0	82	0	0	0	0	1389
7	1	3	–	–	–	–	–	–	–	–	–	–	–	–	–	–	–	–	–	–	2019
Total			724	412	749	292	310	515	1	0	0	0	0	0	0	1745	1507	0	704	19	34165

a Level 1: well-established pharmacovigilance; level 2: partially implemented pharmacovigilance; level 3: basic or no pharmacovigilance

b During the 2021 pandemic, all cases of COVID-19 AEFI were treated exclusively in hospitals, including UHCs, and were reported first to the health administrative region and then forwarded to the CAPM, where they were entered into a specific database

c A total of 1487 AEFI cases were collected in the UHC of Fez during an active surveillance following COVID-19 vaccination of healthcare workers

## Key Points

This paper provides insights into the pharmacovigilance activities of seven university hospital centers (UHCs), given their position as the main providers of healthcare and key sources for reporting serious adverse events in Morocco.

The World Health Organization provides standardized pharmacovigilance indicators and a Global Benchmarking Tool to allow national health authorities to objectively assess and structure their pharmacovigilance and regulatory systems.

The three highest-performing UHCs in Morocco were distinguished by advanced levels of pharmacovigilance activities such as signal detection and validation, as well as decision-making following adverse event concerns. A collaborative approach was used to generate buy-in to formalize pharmacovigilance across all seven centers.

## Footnotes

1 CAPM, in collaboration with Rabat Collaborating Center, organizes a yearly inter-country pharmacovigilance course that has trained, over 15 years, hundreds of national and international health professionals, especially from Sub-Saharan countries (12). This online training takes place in three modules over 2 weeks each, with the objective of developing pharmacovigilance knowledge, know-how, and the ability to set up and manage a pharmacovigilance center (https://www.rcc-pv.org/).
