# Supplementary material for: Assessment of Pharmacovigilance Across University Hospitals in Morocco
Source: Drug Saf. 2025 Feb 12;48(5):527–39. doi: 10.1007/s40264-025-01517-w (PMC11981840; doi:10.1007/s40264-025-01517-w)
Supplement: Supplementary file 1 — Supplementary file1 (PDF 374 KB) [file 40264_2025_1517_MOESM1_ESM.pdf]

Supplementary material N°1

Title: Assessment of pharmacovigilance across university hospitals in Morocco

Journal: Drug Safety

Authors:

Hind Hamzaoui[1], Anna Shaum[2], Imad Cherkaoui[3], Latifa Ait Moussa[1], Houda Sefiani1, Ismail Talibi[1], Ghita Benabdallah[1], Omar Salman[2], Seth Ferrey[2] and Rachida Soulaymani Bencheikh[1]

Affiliations:

1. Centre Anti Poison et Pharmacovigilance du Maroc, Ministry of Health, Rabat, Morocco
2. US Centers for Disease Control and Prevention, Atlanta, USA
3. Private consultant, Morocco

Correspondence:

Rachida Soulaymani Bencheikh: r.soulaymani@pharmacovigilance.ma

ABBREVIATIONS

ADR	Adverse Drug Reaction
AE	Adverse Event
AEFI	Adverse Event Following Immunization
CAPM	Centre Anti Poison et de Pharmacovigilance du Maroc (PV central level)
CPV	Correspondent pf Pharmacovigilance
DTC	Drug and Therapeutics Committee
FP	Focal Point
ICU	Intensive Cre Unit
IEC	Information, Education and Communication
MD	Medical Device (Adverse Event)
ME	Medical Error
NND	National Notification Days
PV	Pharmacovigilance

Vigilance Field Visit Assessment Questionnaire at University Hospital Level

General information

- **Institution(s) assessed:**
- **Persons met and interviewed:**
- **Date of interview:**

Question No.	Question	Response (closed-ended)	Response (open-ended)	Comments/Notes
**SYSTEMS, STRUCTURE, RESSOURCES AND STAKEHOLDERS**				
1	Are you aware of the national guidelines (regulations, guidelines and collaboration agreement) for pharmacovigilance (PV) ?	Response:<br>☐ YES<br>☐ NO<br><br>IF YES<br>• Specify which guidelines<br>☐ Good practice of PV<br>☐ Adverse event following immunization (AEFI) surveillance guidelines<br>☐ Circular of PV<br>☐ Circulars relating to the PV of vaccines against COVID-19.<br>☐ Collaboration agreement between your university hospital and the CAPM (2018)	Briefly describe their contents/terms of reference<br><br>Specify the impact of the collaboration agreement on PV activities in the hospital	
2	To which staff levels have these guidelines for PV been communicated?	Select all that apply<br>☐ Drug and Therapeutics Committee (DTC)<br>☐ Correspondents<br>☐ Local PV focal point<br>☐ Physicians<br>☐ Nurses in hospitals<br>☐ Pharmacists in hospitals		

**3**	**Is PV included within the annual strategic operating plan of your hospital?**	**Response:**<br>☐ **YES**<br>☐ **NO**<br><br>**If YES, can you provide a copy of the terms of reference?**<br>☐ **YES**<br>☐ **NO**		
**4**	**Is there an annual budget component specific to the PV in your hospital?**	**Response:**<br>☐ **YES**<br>☐ **NO**	**If YES, Specify the global amount for the PV**	
**5**	**Is there a structure specifically dedicated to PV activity in the hospital?**<br><br>**How many person-days work in the PV structure?**<br><br>**Is there a designated PV correspondent (CPV) within the hospital?**	**Response:**<br>☐ **YES**<br>☐ **NO**<br><br>**If NO, if there is a PV activity located in a department or clinical service of the hospital, specify which one:**<br>☐ **Central Pharmacy**<br>☐ **Clinical Services**<br>☐ **Other**<br><br>**If NO, if there is a PV activity located outside the hospital within the university hospital, to be specified in memo**<br><br>**Response:**<br>☐ **YES**<br>☐ **NO**	**If YES, specify if the activity appears in the organization chart of the Hospital**<br><br>**Specify the organization outside the hospital**<br><br>**Specify number ________persons**<br>**Specify the number of working days per week dedicated to PV per person _______________**	

		If YES; specify the qualification of the CPV<br>☐ Doctor<br>☐ Pharmacist<br>☐ Nurse<br>☐ Others specify in memo<br><br>If YES: is it the same person who is in charge of all vigilance (materiovigilance, vaccinovigilance, quality defect, etc.)<br>☐ YES<br>☐ NO<br><br>If YES, can you provide us with the terms of reference and contact details of the CPV<br>☐ YES<br>☐ NO	If NO, detail in memo	
	Are there PV representatives (focal points) within the departments and services of the hospital, appointed by the hospital structure or outside?	Response:<br>☐ YES<br>☐ NO<br><br>If YES, can you provide the TOR and coordinates of PV focal points<br>☐ YES provide the documents<br>☐ NO<br>Specify their qualifications:<br>☐ Doctor<br>☐ Pharmacist<br>☐ Nurse<br>☐ Head nurse<br>☐ Other, specify	How many PV focal points (FP) in your hospital?<br>______FP<br>List the clinical services or departments concerned<br><br>If YES, Specify the number of clinical departments or services with a focal point out of the total number of departments or services,	

	**Is there a general procedure for operating PV in your hospital?**<br><br>**Does it have material resources dedicated to PV?**	**Response:**<br>☐ **YES**<br>☐ **NO**<br><br>**Response:**<br>☐ **YES**<br>☐ **NO**<br><br>**If YES, Specify**<br>☐ **Computer**<br>☐ **Means of communication**<br>☐ **Internet connection**<br>☐ **Library**<br>☐ **Other reference sources for drug safety information to be specified in memo**	**Provide procedure**	
**6**	**Do you have the necessary tools for the operation of pharmacovigilance?**	**Specify all that apply:**<br>☐ **ADR/AEFI notification form**<br>☐ **WHO AEFI Case Investigation Form**<br>☐ **Access to VigiFlow**<br>☐ **Other…**	**Specify in memo:**	
**7**	**Detail the functioning of the PV system within the hospital (collaboration between the HCW, the PV correspondent, the PV focal points, the DTC, the hospital direction and the patients?**	**Is the reporting structure clear.**<br>☐ **YES**<br>☐ **NO**	**Please provide flowchart or operating circuit**	

**8**	**Is there regular collaboration between the CAPM and your hospital to review safety issues for medicines and other health products?**<br><br>**Is there an established notification circuit for sending AE cases to the CAPM?**<br><br>**What means of notification do you use to transmit cases of PV (EIs, EIPV) at the CAPM level?**	**Response:**<br>☐ **YES**<br>☐ **NO**<br><br>**If YES. check the fields of application**<br>☐ **Sharing of reports of adverse events of drugs and other health products (AEs)**<br>☐ **Sharing of information regarding drug and other health product safety issues**<br>☐ **Sharing analysis reports**<br>☐ **Participation in regular PV meetings: CAPM PV staff**<br>☐ **Participation in meetings of the CAPM PV technical committee**<br>☐ **Other: please specify:**<br><br>**Response:**<br>☐ **YES**<br>☐ **NO**<br><br>**Select the means used:**<br>☐ **Notification sheet for EIs (yellow yellow sheet)**<br>☐ **EIPV notification form**<br>☐ **VigiFlow**<br>☐ **Electronic notifications (e-reporting)**<br>☐ **SMS**<br>☐ **Email,**<br>☐ **Excel**<br>☐ **WhatsApp**<br>☐ **PV Staff**<br>☐ **Other, specify:**		

	What means of notification did you use to transmit cases of AEFI at the national level during the anti-COVID-19 vaccination campaign?	Select all related:<br>☐ AEFI notification form<br>☐ Specific notification form for AEFIs related to COVID-19 vaccines<br>☐ Mobile app (yakadaliqah)<br>☐ Vigiflow<br>☐ Electronic register (Excel)<br>☐ SMS,<br>☐ Email,<br>☐ WhatsApp<br>☐ Other specify:		
	Do you know the notification deadlines for AEs/AEFIs/MDs specified in the national guidelines?	Response:<br>YES: ☐ ADR ☐ AEFI ☐ MD<br>NO: ☐ ADR ☐ AEFI ☐ MD<br><br>If YES, do you follow them?<br>☐ YES<br>☐ NO<br><br>If NO, specify the notification periods used:<br>For severe ADR<br>☐ As soon as it occurs<br>☐ Weekly<br>☐ Monthly<br>☐ Quarterly<br>☐ Half-yearly<br>☐ Annual<br>☐ Other, specify:<br><br>For serious AEFI<br>☐ As soon as it occurs<br>☐ Weekly<br>☐ Monthly<br>☐ Quarterly<br>☐ Half-yearly<br>☐ Annual<br>☐ Other, specify:	Notification deadlines<br>If YES, briefly describe these delays (in hours or days)<br>- Severe ADR: __________<br>- Non-serious ADR: _______<br>- Incidents related to medical devices: ________<br>- Severe AEFI: _______	

9	Is there regular collaboration between the National Immunization Program (EPI) and your hospital to analyze vaccine safety issues?	Response:<br>☐ YES<br>☐ NO<br><br>If YES, check the fields of application<br>☐ Training on new vaccines<br>☐ Reporting of AEFI<br>☐ Investigation of AEFI<br>☐ Sharing report analysis<br>☐ Other: specify:		
10	Was the hospital PV unit reinforced during the vaccination campaign against COVID-19 ?	Response:<br>☐ YES<br>☐ NO<br><br>If YES, specify which ones:<br>☐ Human resources<br>☐ Material resources<br>☐ Procedures<br>☐ Other : specify:		
11	What types of vaccines are provided/administered in your hospital?	Specify vaccines:<br>☐ COVID-19<br>☐ Hepatitis B Birth dose		
12	Does the hospital have a functional Drug and Therapeutics Committee (DTC) or equivalent?	Response:<br>☐ YES<br>☐ NO<br><br>If YES, can you provide a copy of the terms of reference?<br>☐ YES<br>☐ NO	If YES, specify the main types of activity	

	**Is the PV activity included in the missions of this committee?**	**Response:**<br>☐ **YES**<br>☐ **NO**<br><br>**If YES, can you provide us with a copy of the minutes of the last meetings and/or the PV statistics of the previous year (January 2021-December 2021)**		
	**Is the PV correspondent part of this committee?**	**Response:**<br>☐ **YES**<br>☐ **NO**		

Question No.	Question	Response (closed-ended)	Response (open-ended)	Comments/Notes
		**DETECTION, REPORTING AND DATA MANAGEMENT**		
13	How are suspected ADR or AEFI reported within your hospital?	Specify all that apply:<br>☐ Paper notification form<br>☐ Hospital website<br>☐ Phone/SMS<br>☐ WhatsApp<br>☐ Emailing<br>☐ Internal electronic communication application<br>☐ Register of AEs (electronic/paper).<br>☐ Vigiflow<br>☐ Other, specify:		
14	In the event that the AE notification tool is the paper form?	For ADR, specify the sheet used:<br>☐ standard national declaration form (yellow notification form)<br>☐ Other paper form<br><br>For AEFI, specify the form used:<br>☐ standard national declaration form (yellow notification form)<br>☐ Specific WHO AEFI sheet with the 25 basic variables<br>☐ Other paper form	If other paper form, does the tool include all variables required in the notification (ask to see a copy for each)	
15	Which kind of medical product or vaccine-related problems are reportable at your hospital?	Specify all that apply:<br>☐ Suspected ADR<br>☐ Suspected AEFI<br>☐ Lack of effect/ therapeutic failure<br>☐ Quality problem<br>☐ Medication errors/Immunization errors<br>☐ Drug addiction<br>☐ Overdose<br>☐ Incidents related to medical devices<br>☐ Other, specify:		

16	Who can report AE in your hospital?	Specify all that apply:<br>☐ Doctors<br>☐ Pharmacists<br>☐ Nurses<br>☐ Assistant nurses<br>☐ Patients/Public<br>☐ Other, specify:		
17	How many PV cases were notified by the hospital, by service or department, during the previous year (Jan - Dec 2021)?		Provide statistics, if available, preferably specify<br>By department:<br>- Pediatrics: _____<br>- ICU: _____<br>- Emergencies: _____<br>- Day consultation: _____<br>- Dermatology: _____<br>- Other specify:<br><br>By category :<br>- ADR: _____<br>- AEFI: _____<br>- ME: _____<br>- Quality issues: _____<br>- Medical devices: _____<br>- Misuse, overdose: ____<br>- Other, specify:	
18	What is the percentage of PV cases transmitted to the CAPM?		Specify the %:	
19	Do you have a database or archive of PV cases at your hospital level?	Response:<br>☐ YES<br>☐ NO<br><br>If YES, specify which one :<br>☐ Excel<br>☐ Vigiflow<br>☐ Paper-based register<br>☐ Other, specify:		

	**Who is in charge of entering PV cases?**	**Select one or more answers:**<br>☐ **PV correspondent**<br>☐ **PV structure team**<br>☐ **PV Focal Point of clinical service**<br>☐ **Health Professionals**<br>☐ **Other, specify:**		
**20**	**How many total cases are there in the database for the years 2020-2022?**<br><br>**Of the total, how many are AEFIs?**		**Provide statistics if possible:**<br>- **Total AE cases in 2020: _____**<br>- **AEFI in 2020: _____**<br><br>- **Total AE cases 2021: _____**<br>- **AEFI in 2021: _____**<br><br>- **Total AE cases in 2022: _____**<br>- **AEFI in 2022: _____**	
**21**	**Did you participate in the national PV case notification days (NND) of 2016 and 2018, organized by the CAPM?**<br><br>**Do you recommend doing this day annually?**	**Response:**<br>☐ **YES**<br>☐ **NO**<br><br>**Response:**<br>☐ **YES**<br>☐ **NO**	**If NO, please explain why**<br><br>**If YES, how many cases did you collect during these days compared to all the cases received during the year**<br>- **Total cases in 2016: _____**<br>- **Nb of cases during NND in 2016: _____**<br><br>- **Total cases in 2018: _____**<br>- **Nb of cases during NND in 2018: _____**	

Question No.	Question	Response (closed-ended)	Response (open-ended)	Comments/Notes
**ANALYSIS OF AEFI CASES**				
22	Do you carry out an investigation for serious cases of AEFI? (this question only concerns vaccines and not drugs)	Response:<br>☐ YES<br>☐ NO	If YES, present an example of an investigation carried out	
23	Does the hospital have a vaccine safety surveillance committee responsible for oversight, investigation of AEFIs and causality assessment ?	Response:<br>☐ YES<br>☐ NO<br><br>If YES, can you provide the ToR and the members of this committee?<br>☐ YES<br>☐ NO	If NO, explain how you investigate an AEFI case<br><br>If NO, how do you analyze cases of EIPV?	
24	Do you have standard operating procedures (SOPs) for investigation ?	Response:<br>☐ YES<br>☐ NO	If YES, please provide documents	Add written to each procedure
25	What is the total number of investigations performed in your hospital during the previous year (Jan-Dec 2021)??		Specify the total number:	
26	Do all PV case investigation reports get transmitted to the CAPM ?	Response:<br>☐ YES<br>☐ NO	If NO, specify the number of investigation reports transmitted in 2021	
27	Have all investigated cases undergone a causality assessment?	Response:<br>☐ YES<br>☐ NO	If NO, specify the number of cases assessed	

Question No.	Question	Response (closed-ended)	Response (open-ended)	Comments/Notes
**ANALYSIS OF ADR CASES**				
**28**	**Is the imputability study of drug-related ADRs carried out within your hospital?**	**Response:**<br>☐ **YES**<br>☐ **NO**	**If YES, what methods do you use?**	
**29**	**Is the assessment of the seriousness of adverse drug reactions carried out within your hospital?**	**Response:**<br>☐ **YES**<br>☐ **NO**	**If YES, according to what criteria**	
**30**	**What is the number of drug-related ADR cases analyzed in 2021?**		**Specify the total number:**	
**31**	**Do you organize dedicated staff to analyze cases of adverse events related to medicines and health products?**	**Response:**<br>☐ **YES**<br>☐ **NO**	**If yes, please specify action taken**	

Question No.	Question	Response (closed-ended)	Response (open-ended)	Comments/Notes
**SIGNAL DETECTION AND EVALUATION**				
32	Do you detect PV signals?	Response:<br>☐ YES<br>☐ NO	If YES:<br>☐ Which method do you use?<br>☐ How many signals were detected in 2021?<br>☐ Which ones?	
33	Do you validate the signals detected at the hospital level?	Specify all that apply, for example,<br>☐ Consultation of literature or national or international databases<br>☐ Use of causal method<br>☐ Discussion in the DTC<br>☐ Discussion with PV Staff<br>☐ No local audit or assessment?	If YES :<br>☐ Describe the process used including collaboration with the CAPM<br>☐ How many signals were validated during 2021?<br>☐ Which ones?	
34	Does the hospital have a committee for the investigation of a PV signal?	Response:<br>☐ YES<br>☐ NO	If YES, give the Terms of reference	
35	Have signals detected and validated over the last three years (2020-2022) in your hospital been the subject of a regulatory decision by the DMP[1] (suspension, recall, update of the product instructions....)?	Response:<br>☐ YES<br>☐ NO	If YES, please specify the measures taken	
36	Have you already decided to set up an active surveillance study following the detection of a signal in your hospital?	Response:<br>☐ YES<br>☐ NO<br><br>If YES, indicate for each study:<br>• The type of study	If YES, please provide documents<br><br>Indicate the number of studies:	

[1] Drug and Pharmacy Direction (MoH)

		☐ **Stimulated spontaneous reporting**<br>☐ **Prospective study**<br>☐ **Case-controls**<br>☐ **Cohort Event Monitoring**<br>☐ **Cohort**<br>☐ **Post-marketing surveillance study**<br>☐ **Other specify:**<br><br>• **And according to the stage of completion**<br>☐ **Started**<br>☐ **Ongoing**<br>☐ **Completed**		

Question No.	Question	Response (closed-ended)	Response (open-ended)	Comments/Notes
TRAINING, INFORMATION, EDUCATION AND COMMUNICATION (IEC) WITH CONCERNED GROUPS				
37	How do you provide feedback to internal notifiers of cases of adverse events related to health products?	Specify all that apply:<br>☐ Acknowledgment (Electronic /paper/verbal, automatic or not<br>☐ Feedback with the request for additional information<br>☐ Feedback with case imputability assessment<br>☐ Feedback with support recommendation<br>☐ No feedback<br>☐ Other, specify:	.	
38	Do you have a communication strategy within the hospital?<br><br>Do you have a communication plan in your hospital?	Response:<br>☐ YES<br>☐ NO<br><br>Response:<br>☐ YES for drug safety<br>☐ YES for vaccine safety<br>☐ YES for other health products<br>☐ NO	If YES, specify the type of document and the hierarchical level(s) to which it(they) apply(s).<br><br>Please attach the document.	
39	How are the mechanisms in place to disseminate PV or medical product safety information to members of staff of your hospital?	Specify all that apply<br>☐ Newsletter<br>☐ Information bulletin<br>☐ Bulletin board<br>☐ Website of the hospital<br>☐ Mobile app<br>☐ Phone line<br>☐ Weekly or monthly meeting<br>☐ Other, specify:		

40	**Has your hospital organized continuing education sessions on PV vaccinevigilance including PV of anti-COVID-19 vaccines in the 2 past yeaSr (2021 -2022)?**	**Response:**<br>☐ **YES**<br>☐ **NO**	**Provide statistics and records on the number:**<br>**• trained staff:**<br>**• organized training sessions:**<br><br>**When was the last training session (including vaccinovigilance)**<br><br>**Date:**	
41	**Has your hospital staff participated in the training sessions and PV scientific days organized by the CAPM during the previous two years (2021-2022)?**	**Response:**<br>☐ **YES**<br>☐ **NO**<br><br>**If no precise data, indicate an estimated proportion of participation::**<br>☐ **<10%**<br>☐ **10 to <25%**<br>☐ **25 to <50%**<br>☐ **50 to <75%**<br>☐ **>=75%**	**If YES, specify the number if possible**<br><br>**• Trained personnel:**<br>**• Training sessions:**	
42	**Have your staff participated in PV training sessions and scientific days organized by the Ministry of Health/your health region in the previous two years (2021-2022)?**	**Response:**<br>☐ **YES**<br>☐ **NO**<br><br>**If NO, indicate an estimated proportion of staff who attended :**<br>☐ **<10%**<br>☐ **10 to <25%**<br>☐ **25 to <50%**<br>☐ **50 to <75%**<br>☐ **>=75%**	**If YES, specify the number if possible**<br><br>**• Trained personnel:**<br>**• Training sessions:**	
43	**Other activities performed in your hospital that relate to PV**	**To specify:**<br>☐ **Scientific publications**<br>☐ **Participation in meetings (seminar, congress, …)**<br>☐ **Thesis**		
