# Supplementary material for: Assessment of Pharmacovigilance Across University Hospitals in Morocco
Source: Drug Saf. 2025 Feb 12;48(5):527–39. doi: 10.1007/s40264-025-01517-w (PMC11981840; doi:10.1007/s40264-025-01517-w)
Supplement: Supplementary file 2 — Supplementary file2 (PDF 255 KB) [file 40264_2025_1517_MOESM2_ESM.pdf]

Supplementary material N°2

Title: Assessment of pharmacovigilance across university hospitals in Morocco.

Journal: Drug Safety

Authors:
Hind Hamzaoui[1], Anna Shaum[2], Imad Cherkaoui[3], Latifa Ait Moussa[1], Houda Sefiani1, Ismail Talibi[1], Ghita Benabdallah[1], Omar Salman[2], Seth Ferrey[2] and Rachida Soulaymani Bencheikh[1]

_**Affiliations:**
1. Centre Anti Poison et Pharmacovigilance du Maroc, Ministry of Health, Rabat, Morocco
2. US Centers for Disease Control and Prevention, Atlanta, USA
3. Private consultant, Morocco

Correspondence:
Rachida Soulaymani Bencheikh: r.soulaymani@pharmacovigilance.ma

Table 6: Summary table of the assessment of pharmacovigilance systems at 7 University Hospital Centers with 30 hospitals, Morocco, January 2023

**Implementation level**	**Graduation**
Activity implemented	Rate=3
Activity PARTIALLY implemented	Rate=2
Activity BEING implemented	Rate=1
Activity NOT implemented	Rate=0

**SITE**			**UHC SCORE**	**28 STRATEGIC PHARMACOVIGILANCE (PV) ACTIVITIES**																											
				**Systems, structures and stakeholders**												**Detection, reporting...**						**Case analysis**						**IEC[a]**			
Assessed UHC code (N= 7)	Number of hospitals (N=30)	Hospital number	Implementation rate of PV activities	Guidelines Communication	PV within hospital plan	PV budget	Dedicated structure/full-.	Correspondent of PV	Focal points in clinical .	PV Procedure	Tools (forms / VigiFlow®)	Hierarchy of PV system	Collaboration with CAPM[b]	Hospital committee	PV Activity in committee	Notification tools	PV problems notification	Notifiers	100% Notification to CAPM	Database and statistics	National Notification Days	Imputability (ADR[c])	Seriousness (ADR	Case analysis Staff (ADR)	Investigation of AEFI[d]	*Validation of signals	Decisions after signals	Feedback	Dissemination mechanisms	Hospital training	CAPM training (21/22)
1	10	1	**94%**																												
		2	**83%**																												
		3	**65%**																												
		4	**50%**																												
		5	**45%**																												
		6	**35%**																												
		7	**30%**																												
		8	**23%**																												
		9	**15%**																												
		10	**4%**																												
2	4	11																													
		12	**85%**																												
		13																													
		14	**32%**																												

a. Information, Education and Communication
b. Centre Anti Poison et de Pharmacovigilance du Maroc (PV central level)
c. Adverse Drug Reaction
d. Adverse Event Following Immunization

Table 6: (continued): Summary table of the assessment of pharmacovigilance systems at 7 University Hospital Centers with 30 hospitals, Morocco, January 2023

Implementation level	Graduation	Implementation level	Graduation
Activity implemented	Rate=3	Activity BEING implemented	Rate=1
Activity PARTIALLY implemented	Rate=2	Activity NOT implemented	Rate=0

SITE			UHC SCORE	28 STRATEGIC PHARMACOVIGILANCE (PV) ACTIVITIES																											
				Systems, structures and stakeholders												Detection, reporting...						Case analysis						IEC[a]			
Assessed UHC code (N= 7)	Number of hospitals (N=30)	Hospital number	Proportion of Implemented strategic PV activities	Guidelines Communication	PV within hospital plan	PV budget	Dedicated structure/full-	Correspondent of PV	FP in clinical services	PV Procedure	Tools (forms / VigiFlow®)	Hierarchy of PV system	Collaboration with CAPM[b]	Hospital committee	PV Activity in committee	Notification tools	PV problems notification	Notifiers	100% Notification to CAPM	Database and statistics	National Notification Days	Imputability (ADR[c])	Seriousness (ADR	Case analysis Staff (ADR)	Investigation of AEFI[d] cases	Validation of signals	Decisions after signals	Feedback	Dissemination mechanisms	Hospital training	CAPM training (21/22)
3	5	15	**53%**	0	3	0	0	3	0	0	2	3	0	3	3	3	2	3	0	3	3	3	3	3	0	0	0	2	0	0	3
		16		0	3	0	0	3	0	0	2	3	0	3	3	3	2	3	0	3	3	3	3	3	0	0	0	2	0	0	3
		17		0	3	0	0	3	0	0	2	3	0	3	3	3	2	3	0	3	3	3	3	3	0	0	0	2	0	0	3
		18		0	3	0	0	3	0	0	2	3	0	3	3	3	2	3	0	3	3	3	3	3	0	0	0	2	0	0	3
		19		0	3	0	0	3	0	0	2	3	0	3	3	3	2	3	0	3	3	3	3	3	0	0	0	2	0	0	3
4	5	20	**82%**	3	3	0	0	3	3	2	2	3	3	3	2	3	3	3	3	3	3	3	3	0	0	3	3	3	3	3	3
		21		3	3	0	0	3	3	2	2	3	3	3	2	3	3	3	3	3	3	3	3	0	0	3	3	3	3	3	3
		22		3	3	0	0	3	3	2	2	3	3	3	2	3	3	3	3	3	3	3	3	0	0	3	3	3	3	3	3
		23		3	3	0	0	3	3	2	2	3	3	3	2	3	3	3	3	3	3	3	3	0	0	3	3	3	3	3	3
		24		3	3	0	0	3	3	2	2	3	3	3	2	3	3	3	3	3	3	3	3	0	0	3	3	3	3	3	3
5	4	25	**74%**	3	2	0	0	3	0	3	3	3	3	3	2	3	3	3	2	2	3	3	3	0	3	0	3	3	3	0	3
		26		3	2	0	0	3	0	3	3	3	3	3	2	3	3	3	2	2	3	3	3	0	3	0	3	3	3	0	3
		27		3	2	0	0	3	0	3	3	3	3	3	2	3	3	3	2	2	3	3	3	0	3	0	3	3	3	0	3
		28	**70%**	3	2	0	3	3	0	3	2	3	3	3	3	3	2	3	3	2	0	3	3	2	0	3	0	3	3	3	0
6	1	29	**40%**	0	1	0	1	3	0	0	2	2	0	1	0	3	2	3	3	2	0	3	3	0	0	0	0	3	2	0	0
7	1	30	**29%**	1	1	0	0	3	0	1	1	1	1	1	0	1	1	1	1	1	3	1	0	0	0	0	0	1	1	3	0

a. Information, Education and Communication
b. Centre Anti Poison et de Pharmacovigilance du Maroc (PV central level)
c. Adverse Drug Reaction
d. Adverse Event Following Immunization
